# Characterization of the Long Terminal Repeat of the Endogenous Retrovirus-derived microRNAs in the Olive Flounder

**DOI:** 10.1038/s41598-019-50492-7

**Published:** 2019-09-30

**Authors:** Hee-Eun Lee, Ara Jo, Jennifer Im, Hee-Jae Cha, Woo-Jin Kim, Hyun Hee Kim, Dong-Soo Kim, Won Kim, Tae-Jin Yang, Heui-Soo Kim

**Affiliations:** 1 Department of Integrated Biological Science, Pusan National University, Busan, 46241 Republic of Korea; 2 Institute of Systems Biology, Pusan National University, Busan, 46241 Republic of Korea; 3 Department of Parasitology and Genetics, Kosin University College of Medicine, Busan, 49267 Republic of Korea; 4 Biotechnology Research Divisions, National Fisheries Research and Development Institute, Busan, 46083 Republic of Korea; 5 Departments of Life Science, Sahmyook University, Seoul, 01795 Republic of Korea; 6 Chromosome Research Institute, Sahmyook University, Seoul, 01795 Republic of Korea; 7 Department of Marine Bio-Materials & Aquaculture, Pukyong National University, Busan, 48513 Republic of Korea; 8 School of Biological Sciences, Seoul National University, Seoul, 08826 Republic of Korea; 9 Department of Plant Science, Plant Genomics and Breeding Institute, Research Institute for Agriculture and Life Sciences, College of Agriculture and Life Sciences, Seoul National University, Seoul, 08826 Republic of Korea; 10 Department of Biological Sciences, College of Natural Sciences, Pusan National University, Busan, 46241 Republic of Korea

**Keywords:** Chromosomes, miRNAs, Transcriptional regulatory elements

## Abstract

Endogenous retroviruses (ERVs) have been identified at different copy numbers in various organisms. The long terminal repeat (LTR) element of an ERV has the capacity to exert regulatory influence as both a promoter and enhancer of cellular genes. Here, we describe olive flounder (OF)-ERV9, derived from chromosome 9 of the olive flounder. OF-ERV9-LTR provide binding sites for various transcription factors and showed enhancer activity. The OF-ERV9-LTR demonstrates high sequence similarity with the 3′ untranslated region (UTR) of various genes that also contain seed sequences (TGTTTTG) that bind the LTR-derived microRNA(miRNA), OF-miRNA-307. Additionally, OF-miRNA-307 collaborates with transcription factors located in OF-ERV9-LTR to regulate gene expression. Taken together, our data facilitates a greater understanding of the molecular function of OF-ERV families and suggests that OF-miRNA-307 may act as a super-enhancer miRNA regulating gene activity.

## Introduction

*Paralichthys olivaceus*, known as olive flounder (OF) is an economically important marine flatfish which is extensively cultured in Korea, China and Japan. Due to their high economic value, there are several selective breeding programs in place, such as those involving sex manipulation, owing to differences in growth speed and size between male and female olive flounders^1–3^. However, due to farming conditions, olive flounders are prone to fatal infections or diseases caused by various bacteria, viruses and parasites^4–7^. The olive flounder RNA sequencing program has provided important information regarding known alternative splicing patterns and gene duplication events within the olive flounder^8^. However, there are few studies which characterize olive flounder from a molecular biology approach.

Endogenous retroviruses (ERVs) are one of the transposable elements (TE) which are inherited as stable genomic components throughout the evolution of a species. Depending on the species, copy number and chromosomal distribution may vary^9^. ERVs mediate structural variation and genomic instability based on their copy number and reverse transcriptase activity^10^. ERV elements are highly defective, containing large deletions, stop codons, and frameshifts in their open reading frames (ORFs). Moreover, structural genes from some ERV families are preferentially expressed in various tissues and cancer cell lines^11,12^. Multiple-copy of ERV families scattered throughout the genome have been reported to regulate the expression of neighbouring genes^13–15^. Long terminal repeat elements (LTR) recruit transcription factors and thus can enhance the transcription of host cell genes^11,15^. LTR elements may contain intrinsic enhancer activity; however, base substitutions, transcription factor binding sites (TFBS), bi-directional transcription start sites (TSS), and open chromatin may increase the enhancer activity of LTR elements^16^. Tissue-specific promoter and enhancer activity of human endogenous retrovirus (HERV) -K LTR has been shown in several human and CHO cell lines^17^. Furthermore, studies have shown that species-specific ERV enhancer activity is generally restricted to hypomethylated tissues, suggesting that ERV families are an important mediator in evolutionary regulation^18^. In the case of olive flounder, chromosome 5-derived OF-ERV5-LTR has shown promoter activity in HepG2 and HINAE cells which prompted us to investigate the role of OF-ERV9-LTR in this study^19^.

MicroRNAs (miRNAs) are small noncoding RNA molecules made up of 19 to 22 nucleotides (nt), that play important roles in gene regulation^20–22^. Primary miRNAs (pri-miRNAs) are a long double-stranded RNA (dsRNA) transcript with a hairpin structure, which is identified by the nuclear protein DiGeorge Syndrome Critical Region 8 (DGCR8). The RNA-binding protein DGCR8 together with the RNase III enzyme Drosha recognize and cleave the hairpin RNA, thereby turning pri-miRNA into pre-miRNA. The formed pre-miRNA also adopts a hairpin structure which is processed into mature miRNAs by Dicer. Individual miRNAs bind to 3′ untranslated region (UTR) of target gene through a critical region called the “seed region”, in order to regulate gene expression by either translational repression or mRNA degradation^20^. Previously, TE-derived human miRNA genes were discovered, and it was shown that several miRNA genes share their sequences with TEs^23,24^.

In this study, the genomic structure, chromosomal location and enhancer activity of OF-ERV9-LTR have been analysed. The enhancer activity of OF-ERV9-LTR was controlled by TFBSs. In addition, a novel OF-ERV9-LTR-derived miRNA was discovered. The relative expression and functional studies of the OF-ERV9-LTR-derived microRNA-307 (OF-miR-307) suggest that OF-ERV-LTR elements may contribute to several biological functions in olive flounder.

## Results

### Structure and chromosomal location of OF-ERVs

**Figure 1:**
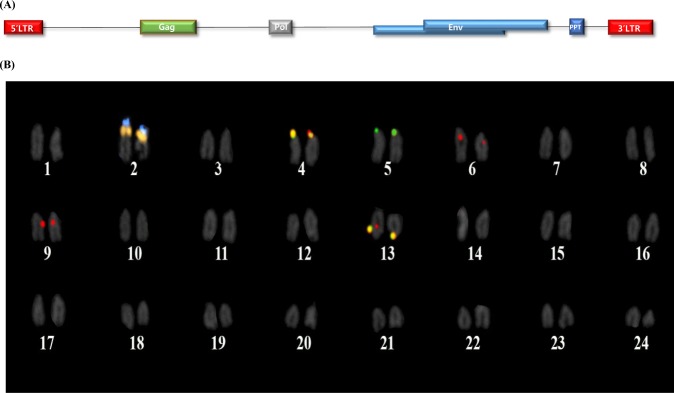
(**A**) The schematic structure of OF-ERV9, created using RetroTector. In addition to a 5ʹ and 3- LTR, this sequence includes genes encoding the gag, pol, env and ppt proteins. (**B**) Karyogram of a female olive flounder showing 5S (blue) and 45S (orange) rDNA, OF-ERV5-LTR (green), and OF-ERV9-LTR (red). Colocalization of two LTR elements is indicated in yellow.

The schematic structure of OF-ERV9 shows that each terminal contains an LTR, with the *gag*, *pol*, *env*, and *ppt* genes placed between them (Fig. 1A). The reference 5S and 45S rDNA repeats were distinctly observed in the metaphase chromosome spread of the olive flounder, with one pair of each 5S and 45S rDNA located side by side on the short arms, near the centromeric region of the sub-telocentric chromosome 2. Three pairs of OF-ERV5-LTR signals were observed on the short arm of chromosome 4 and 5 and on the long arm of chromosome 13 (Fig. 1B). Meanwhile, four pairs of OF-ERV9-LTR signals were observed, which were localized on chromosomes 4, 6, 9 and 13. The signals on chromosome 6 and 9 were detected in the pericentromeric region. In the case of the two homologous chromosome pairs on chromosomes 4 and 13, the yellow signal suggested that OF-ERV5-LTR and OF-ERV9-LTR were located very close to each other, due to the overlap of the green and red signals.

### Sequence analysis of OF-ERV9-LTR and enhancer activity analysis in SW620 cells

**Figure 2:**
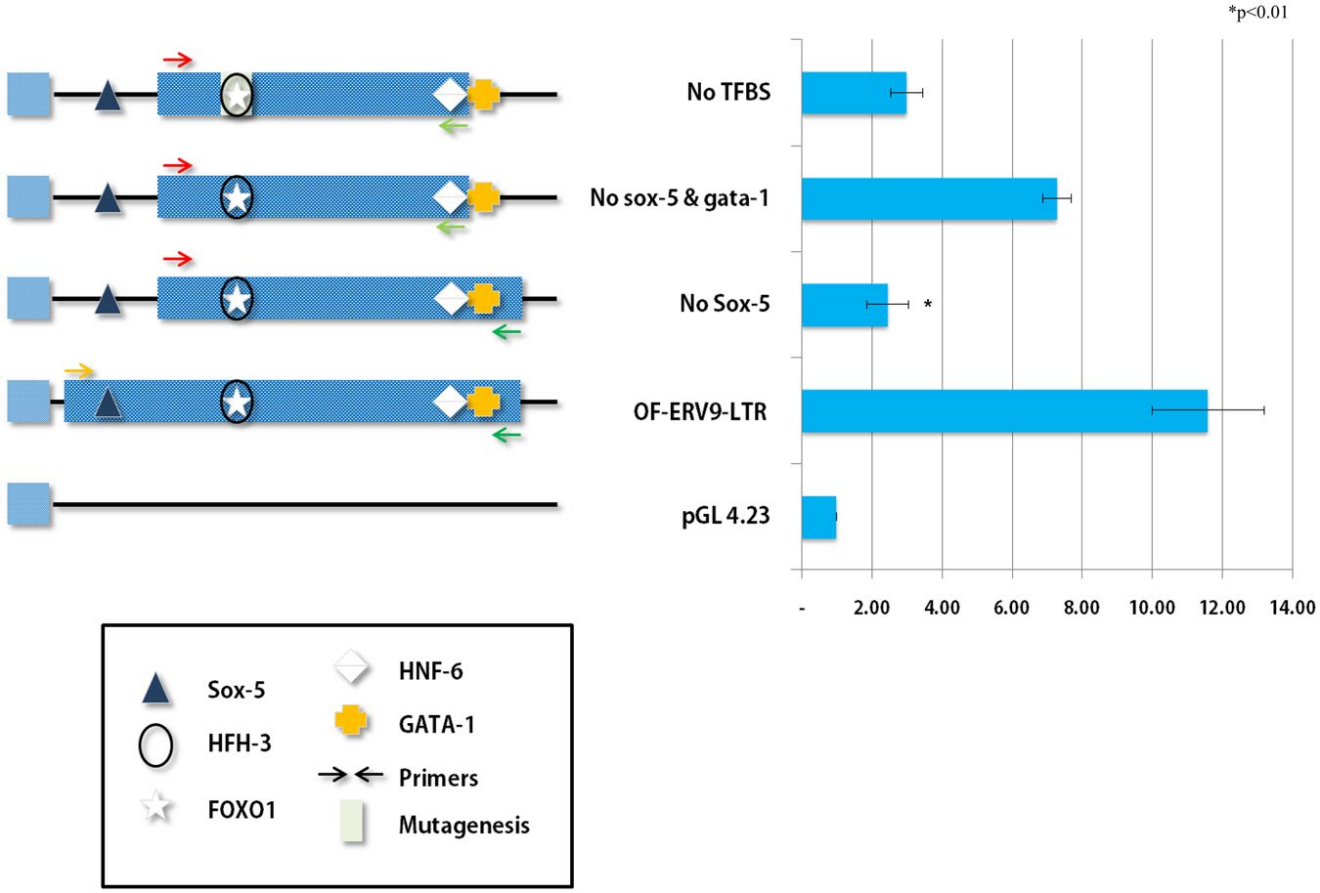
The enhancer activity analysis of OF-ERV9-LTR. Each OF-ERV9-LTR region was cloned into an enhancer vector, as indicated by the schematic structures, and assayed in a cell culture. The structure on the left side describes the vector as well as which transcription factors were cloned. Each arrow represents differently designed primers, described in detail in the Materials and Methods section of the main text. The graph shows the enhancer activity of each cloned plasmid. The data presented represent the mean ± standard error (Student’s t-test vs. control; *p < 0.01).

The TFBSs of OF-ERV9-LTR constructs were analysed by using MATCH in TRANSFAC v8.0. (Suppl Fig. 1). The TRANSFAC program was used to predict TFBSs and those that had a threshold value higher than 0.95 for both core match and matrix match were selected and labelled. Constructs containing the OF-ERV9-LTR region and various permutations were cloned into the pGL-4.23 vector (Promega) and checked for enhancer activity (Fig. 2). The plasmid containing the OF-ERV9-LTR region showed high enhancer activity. This enhancer activity was downregulated when the construct excluded the binding site for the transcription factor (TF) Sox-5. In contrast, the activity increased slightly when Sox-5, GATA-1 and HNF-6 binding sites were excluded. Lastly, point mutations were induced in the binding sites of FOXO1 and HFH-3, enabling the exclusion of all the identified and selected TFBSs. In this instance, enhancer activity was further downregulated.

### Identification and phylogenetic relationship of OF-ERV9-LTR sequences

**Figure 3:**
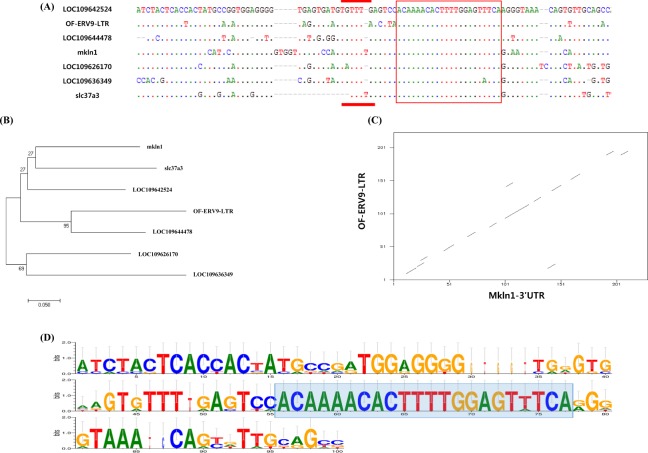
(**A**) OF-ERV9-LTR was analysed in comparison to related target gene sequences. The red line denotes the seed region of OF-miRNA-307 and the red box indicates the conserved region of all genes. (**B**) Phylogenetic tree comparing OF-ERV9-LTR and related genes. Scale bar = 0.05. (**C**) Dot plot comparison of the OF-ERV9-LTR and *mkln1* 3ʹ-UTR sequences. The x axis represents the 3ʹ-UTR of the *mkln1* gene and the y axis represents the LTR of OF-ERV9. (**D**) Sequence alignment of OF-ERV9-LTR and its related genes using the Web Logo software. The blue box indicates the conserved region.

**Figure 4:**
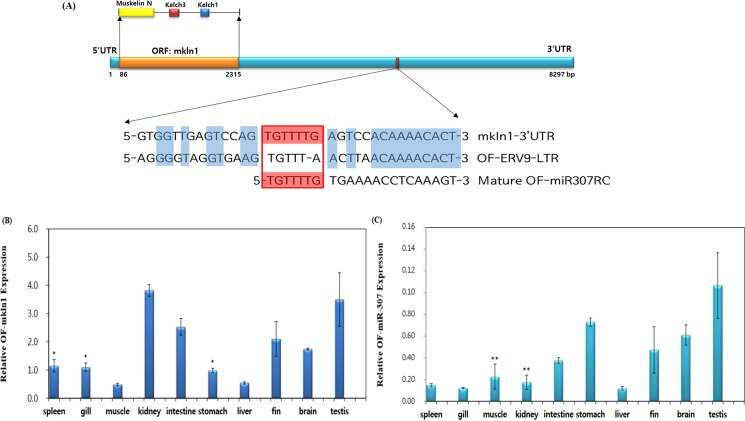
(**A**) Schematic structure of the *mkln1* gene. The Muskelin N, Kelch3 and Kelch1 proteins are contained in the ORF region of the *mkln1* gene. The sequences coloured in blue are conserved regions and the red box is the seed region of the OF-miRNA-307. (**B**) Relative quantitative PCR expression patterns for *mkln1* in various tissues. Amongst all the olive flounder tissues tested, the kidney, intestine, fin and testis showed the highest expression levels of *mkln1*. (**C**) Relative quantitative PCR expression patterns for OF-miRNA-307. Of all the tissues tested, the lowest expression of OF-miRNA-307 was found in the spleen, gills, kidneys and liver. The data presented represent the mean ± standard error (Student’s t-test vs. control; *p > 0.15, **p > 0.02).

OF-ERV9-LTR sequences were analysed using the NCBI database to identify genes with similar sequences. As a result, the olive flounder genes *mkln1*, *slc37a3*, LOC109642524, LOC109644478, LOC109626170, and LOC109636349 were matched with the OF-ERV9-LTR sequence. The identified conserved region is highlighted by a red box in Fig. 3A. A new miRNA, OF-miRNA-307 (LC333100), was identified in the conserved region of the 3ʹ-UTR of *mkln1* and OF-ERV9-LTR (Fig. 4A). The red box in Fig. 4A indicates the seed region of miRNA-307, which binds to the 3ʹ-UTR of both *mkln1* and OF-ERV9-LTR. This region is also indicated in Fig. 3A by a red line. The sequence alignment of OF-ERV9-LTR, *mkln1*, *slc37a3*, LOC109642524, LOC109644478, LOC109626170 and LOC109636349 was visualized by using WebLogo^25,26^ (Fig. 3D), where the blue box indicates conserved regions. The sequences of all six genes and OF-ERV9-LTR were then used to create a phylogeny tree (Fig. 3B). *Mkln1* shows a close relation to *slc37a3*, followed by LOC10964524. On the other hand, OF-ERV9-LTR has greater homology to LOC109644478. Additional evidence for this similarity was provided by the dottup program, where the long, even central line indicates high similarity between the 3ʹ-UTR of *mkln1* and OF-ERV9-LTR (Fig. 3C).

### RNA hybrid structure of miRNA and its target genes

The conserved sequences of OF-ERV9-LTR, which generated OF-miRNA-307, were hybridized with target genes as shown in Supplementary Fig. 2, and the minimum free energy for hybridization was calculated. The RNAhybrid energy scale for OF-miRNA-307 and LOC109644478 was −17.2 kcal/mol (Suppl. Fig. 2A), for *mkln1* it was −20.2 kcal/mol (Suppl Fig. 2B), for LOC109636349 it was −34.5 kcal/mol (Suppl. Fig. 2C) and for *slc37a3 it* was −17.8 kcal/mol (Suppl. Fig. 2D). The lowest energy scales denote a strong interaction between the two molecules. As *mkln1* showed the second lowest energy scale, *mkln1* was selected for further study. Despite the fact that LOC109636349 has the lowest energy scale, it was not selected since it is uncharacterized and is annotated as a noncoding RNA, while *mkln1* is a protein-coding gene.

### Analysis of OF-miRNA-307 and related miRNAs

The sequence of OF-miRNA-307 was analysed by using the miR-base website in order to examine if there are any related miRNAs. As a result, six related miRNAs with conserved regions were identified (Suppl. Fig. 3). Four of these miRNAs were of human origin, belonging to the miRNA-642 family. The other miRNAs belonged to *Pan troglodytes* and *Gossypium*. Of all six analogous miRNAs, hsa-miRNA-642a-5p-RC and ptr-miRNA-642-RC had the highest degree of homology with OF-miRNA-307. As hsa-miRNA-642a-5p-RC was highly conserved, it was used in order to analyse biological processes, cellular components and molecular functions in Gene Ontology^27^ (Suppl. Fig. 4). Metabolic and cellular processes were the most abundant amongst the analysed results, with cell part, catalytic activity and binding activity also being strongly represented.

### Relative expression analyses of *mkln1* and OF-miRNA-307

The expression of *mkln1* and OF-miRNA-307 in various OF tissues was evaluated by qPCR (Fig. 4B,C). The relative expression of *mkln1* in OF kidneys, intestine, fin, and testis tissues was higher than in other OF tissues (Fig. 4B). On the other hand, the spleen, gills, kidneys, and liver showed low expression of OF-miRNA-307 (Fig. 4C).

### Co-transfection of the *mkln1* 3′-UTR and OF-miRNA-307 mimics

**Figure 5:**
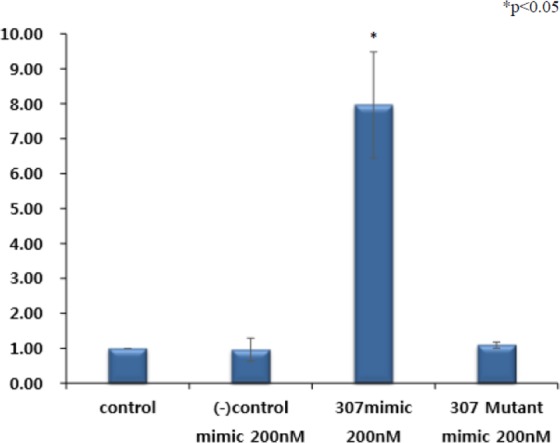
(**A**) Co-transfection of the *mkln1* 3ʹ-UTR and OF-miRNA-307 mimic. In contrast to the negative control and OF-miRNA-307 mimic mutant, co-transfection of the *mkln1* 3ʹ-UTR with the OF-miRNA-307 mimic induced a dramatically increase in expression level. The data presented represent the mean ± standard error (Student’s t-test vs. control; *p < 0.05).

We hypothesized that the co-transfection of *mkln1* 3′-UTR and OF-miRNA-307 mimics in HT-29 cells would result in a lower or similar expression of the OF-miRNA-307 mimic compared to controls and that the OF-miRNA-307 mutant mimic would be overexpressed. However, we found that the OF-miRNA-307 mimic was highly expressed, at a level much greater than the mutant or any of the controls (Fig. 5).

## Discussion

Previous studies regarding olive flounder focused on preventing infections and improving farming conditions, providing valuable outcomes. However, the molecular biological characteristics of olive flounders are still poorly understood. Therefore, the present study aimed to characterize the OF-ERV9-LTR through various bioinformatics tools, in order to provide fundamental information on this subject. As a result, a newly reported miRNA has been identified and described.

ERVs contain enormous deletions, as well as stop codons and frameshifts in their ORFs, and copy number and chromosomal distributions vary between species. TEs insert themselves into any part of the genome, thereby creating novel sequences in the genome which leads to the generation of new genes^9,10,13–15^. The ERV9 found in olive flounder has well conserved LTRs, including *gag*, *pol*, *env* and *ppt* sequences. A previous study has shown that OF-ERV5 also has well-conserved sequences among olive flounder and confirmed the promoter activity of OF-ERV5, 9 and 10 in both HINAE and HepG2 cell lines^19^. The present study is mainly focused on the OF-ERV9-LTR, another highly conserved and widely spread OF-ERV in olive flounder and supports the idea that both TEs truly are “jumping genes”. Alternative promoter and enhancer activity are an additional function of TE, which can act as both a promoter and an enhancer by inserting itself into specific genes^9,28,29^. The present study suggests that OF-ERV9-LTR mainly has enhancer activity, despite having a weak promoter activity^19^. Moreover, TEs provide binding sites for TFs, thereby greatly influencing gene regulation^18,30–32^. It has been previously demonstrated that the tandem repeat region of the LTR12C element is critical for its promoter activity, with or without TFs^33^. Therefore, we decided to eliminate TFBSs nearby OF-ERV9-LTR, based on previous results which suggested that enhancer activity is partially controlled by transcription factors. To examine primary TFs that have enhancer function along with OF-ERV9-LTR, a deletion variant of each TF was designed. Our results suggest that the transcription factor SOX-5 had the strongest enhancer activity when compared with other analysed transcription factors, namely GATA-1, HFH-3, FOXO1 and HNF-6.

The transcription factor SOX-5 is involved in embryonic development, cell fate and differentiation^34,35^. Previous studies have revealed that Sox-5 is an enhancer, as well as a super-enhancer (SE) in a variety of biological processes^36–39^. The transcription factor SOX-5 in OF-ERV9-LTR is located in the region between 166 bp and 170 bp, while the OF-miRNA-307 was located in the region between 103 bp and 108 bp. The close positioning of both Sox-5 and OF-miRNA-307 within the OF-ERV9-LTR can lead to the generation of enhancer RNA (eRNA). One study indicated that eRNA plays a role in regulating transcription and that it may also act as an activator rather than a repressor of the target promoter region^40^.

**Figure 6:**
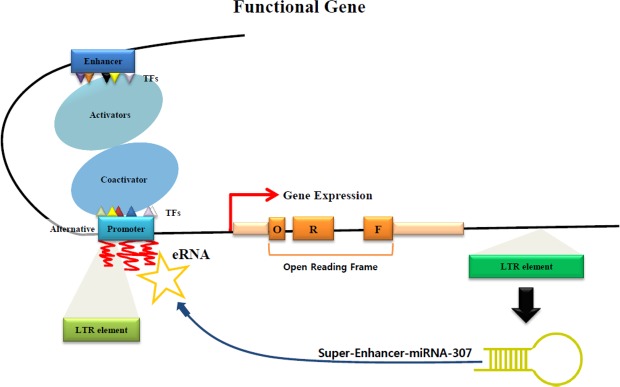
Schematic indicating the predicted roles of OF-miRNA-307. It is possible that OF-miRNA-307 functions as an SE-miRNA and that this SE-miRNA activates the generation of eRNA, resulting in a dramatic increase in the expression of the target gene.

miRNAs derived from TEs have been identified in several studies, which showed that various types of TEs, such as long interspersed elements (LINE), short interspersed elements (SINE), LTRs and DNA transposons, can give rise to several miRNA families^23,24,41,42^. Furthermore, these studies suggest that TEs may have affected mammalian evolution by inducing the creation of new miRNAs. TE-derived miRNAs not only bind to the 3′-UTR of target genes but can also bind to their 5′-UTR^23^. In the case of OF-ERV9-LTR, it can generate both OF-miRNA-307 and the 3′-UTR of the *mkln1* gene (Fig. 4). The OF-ERV9-LTR inserted itself into the olive flounder genome thereby creating the OF-miRNA-307. A few studies have shown that miRNAs that act as an enhancer when binding to the 3ʹ-UTR region of its target gene are called super-enhancer-miRNAs (SE-miRNA)^43,44^. We hypothesize that the OF-miRNA-307 may function as a SE-miRNA by cooperating with the super-enhancer TF SOX-5 (Fig. 6). The OF-ERV9-LTR-derived miRNA, OF-miRNA-307, is located in the region neighbouring the enhancer or super-enhancer TF called SOX-5 and can either act as an enhancer or augment the already existing enhancer function of SOX-5. Subsequently, either the enhancer or SOX-5 recruit activators and co-activators to the promoter region of the functional gene. Meanwhile, SE-OF-miRNA-307 collaborates with either the gene promoter or the alternative promoter generated by the LTR element. As such, with the cooperation of the super-enhancer TF SOX-5, OF-miRNA-307 may function as a SE-miRNA in the *mkln1* gene.

We suggest that OF-miRNA-307 may function as a SE-miRNA, through a collaboration with the TF SOX-5. Analysing and determining the enhancer function of OF-miRNA-307 requires further research. However, some studies have already revealed and predicted that enhancer miRNA may play several crucial roles. Our findings illustrate another example of enhancer miRNA activity. Hence, we presume that OF-ERV9-LTR derived OF-miRNA-307 may function as a SE-miRNA, thereby regulating and enhancing gene transcription.

## Materials and Methods

### Ethical statement

All experiments in this study were carried out in accordance with the guidelines and regulation approved by Pusan National University-Institutional Animal Care and Use Committee (PNU-IACUC).

### Isolation of genomic DNA and RNA from olive flounder

The genomic DNA (gDNA) of olive flounder was extracted from blood by using the DNeasy Blood & Tissue Kit (Qiagen, Valencia, CA) according to the manufacturer’s protocol. Extracted gDNA samples were then used for PCR amplification. Total RNA was extracted from 11 healthy olive flounder tissue samples using TRIzol (Invitrogen, Carlsbad, CA) with RNase-Free DNase (New England Biolabs, Beverly, MA) according to the manufacturer’s protocol. The quantity and quality of each gDNA and RNA sample were determined using an ND-1000 UV-Vis spectrophotometer (NanoDrop, Wilmington, DE). The PrimeScript™ RT reagent Kit with gDNA Eraser (TaKaRa, Japan) was used for cDNA synthesis and gDNA removal from 0.5 µg of each total RNA sample.

### Computational data analysis of the LTR-derived OF-miR-307

OF-ERV9-LTR-derived sequences were retrieved from the NCBI database (https://www.ncbi.nlm.nih.gov/). The structure of OF-ERV9 was drawn based on the program RetroTector^45^. The OF-ERV9 5ʹLTR sequence was analysed with BLAST to discover similar families. Following that, sequence alignments were analysed using BioEdit^46^. All aligned sequences were then used to generate a phylogeny tree using MEGA7^47^. The WebLogo program was used to display the conserved sequences in a graphic view^25,26^. The Dottup program (http://www.bioinformatics.nl/cgi-bin/emboss/dottup) was used to check for analogous regions between the 5ʹLTR of OF-ERV9 and the 3ʹ UTR region of *mkln1*. Prediction of LTR-derived OF-miRNA-307 secondary structures was performed by employing the RNAfold WebServer (http://rna.tbi.univie.ac.at/cgi-bin/RNAWebSuite/RNAfold.cgi), considering both seed region pairing and minimum free energy. In order to predict OF-miRNA-307 targets, OF-miRNA-307 was hybridized with target genes through RNAhybrid in the web program BiBiServ (https://bibiserv.cebitec.uni-bielefeld.de/)^48^. The gene ontology program was used to analyse molecular function, cellular components and biological processes of the miRNA-307 analogous miRNA, hsa-miRNA-642a-5p-RC (http://www.geneontology.org/)^27^.

### Fluorescence ***in situ*** hybridization (FISH) of OF-ERVs in olive flounder

A kidney cell suspension was prepared according to the protocol established by Kim (1982)^49^. Briefly, cells were spread onto pre-cleaned slides in a humid chamber and air-dried. The slides were subsequently fixed with 2% formaldehyde, dehydrated in a graded ethanol series (70%, 90%, and 100%) and air-dried. The FISH procedure was done as described by Waminal (2012)^50^ with small modifications. The typically conserved eukaryote FISH signals of 5S and 45S rDNA^51^ were used as reference signals and labelled by PCR with digoxygenin-11-dUTP and biotin-16-dUTP, respectively. Plasmid DNAs, OF-ERV5-LTR and OF-ERV9-LTR, derived from olive flounder, were denatured by boiling for 5 min and then labelled with digoxygenin-11-dUTP and biotin-16-dUTP through nick-translation (Roche, Germany). Chromosome spread slides were treated with 100 µg/mL RNase A in 2 × SSC for one hour at 37 °C. Slides were incubated in both 0.01 N HCI for 2 min at room temperature and 0.005% diluted pepsin in 0.01 N HCI for 10 min at 37 °C and washed with 2 × SSC twice. Slides were post-fixed in 4% paraformaldehyde solution for 10 minutes, washed in 2 × SSC, dehydrated in a graded ethanol series (70%, 90%, and 100%) and then air-dried.

Digoxigenin-labelled OF-ERV5-LTR and 5S rDNA were detected using monoclonal anti-digoxigenin-fluorescein isothiocyanate (FITC) conjugates (Sigma, USA). Biotinylated OF-ERV9-LTR and 45S rDNA probes were detected using Cy™3-streptavidin conjugates (Zymed, USA). Incubated slides were washed in detection buffer at 37 °C and subjected to dehydration in an ethanol series. Slides were air-dried and counterstained with 1 µg/mL 4′-6-diamidino-2-phenylindole (DAPI) (Roche, Germany) in a Vectashield (Vector Lab, Inc., Burlingame, CA) solution. Then, chromosomes were observed under an Olympus BX53 fluorescence microscope equipped with a Leica DFC365 FS CCD camera, using an oil-immersion lens (×100 magnification). Captured images were processed using Cytovision©/Genus™ ver. 7.2 (Leica Microsystems, Germany). Final image enhancements were done through Adobe Photoshop CC (Adobe Systems, San Jose, CA).

### Cloning of the OF-ERV9-LTR gene

In order to perform gene cloning, primers were designed to amplify chromosome 9 of OF-ERV-LTR as follows: forward: 5ʹ-TGC TGT TGT GTG TTA CTG TGC-3ʹ and reverse: 5ʹ-CAT GAC AAC AAA GGA TGC TCA-3ʹ. Primers for variants of Sox-5 containing deletions designed as follows: 5ʹ-ACT GAT CGA TTT TTC AAA CG-3ʹ as the forward primer and 5ʹ-GCA ATG CTA GCA GAA GAT TA-3ʹ as the reverse primer. The variants of GATA-1, HNF6, HFH-2 and FOXO1 that contained deletions shared the same forward primer with the Sox-5 deleted variant and the reverse primer was designed as 5ʹ-TGA TTT TAA CAT GCA ACC TG-3ʹ. Genomic PCR primers were designed using Primer3^52,53^.

PCR was carried out in a total reaction volume of 25 µL, containing 2.5 µL of 10X PCR buffer, 3 µL of each dNTP (stock concentration, 2.5 µM), 0.1 µL of Ex Taq polymerase (TaKaRa, Japan), 16.4 µL of ddH_2_O, 1 µL of each primer and 1 µL of gDNA. After an initial denaturation step at 95 °C for 5 min, the products were amplified for 30 cycles of 95 °C for 30 sec, 55 °C for 30 sec and 72 °C for 90 sec, with a final elongation step at 72 °C for 5 min. The PCR products were separated on a 1.5% agarose gel, purified with the Labo Pass Kit (Cosmogenetech, Korea), and cloned into the pGL-4.23 vector (Promega, USA). The cloned plasmid products were then isolated with the Hybrid-Q Plasmid mini Kit (GeneAll, Korea) and sequenced by Cosmogenetech.

### Quick change mutagenesis

Quick change mutagenesis (Cosmogenetech, Korea) was used to delete the FOXO1 and HFH-3 transcription factor binding sites from the OF-ERV sequence. These transcription factors share the binding site sequence AAACA, which was mutagenized to AGGCA. For this, Cosmogenetech designed the forward 5ʹ-GAT TTT TCA AAC GTA GGC AAA CAA CAG AAA AAT C-3ʹ and reverse 5ʹ-GAT TTT TCT GTT GTT TGC CTA CGT TTG AAA AAT C-3ʹ primers, which were used for PCR amplification with Taq PCR Mastermix (Cosmogenetech, Korea). The total reaction volume was 50 µL, containing 0.5 µL of polymerase, 5 µL of dNTPs (2.5 mM each), 10 µL of 5X buffer, 23.5 µL of ddH_2_O, 5 µL of each primer (10 pmol) and 1 µL of template DNA. The initial denaturation step was held at 95 °C for 5 min, prior to amplification for 20 cycles at 95 °C for 1 min, 55 °C for 1 min and 72 °C for 4 min, followed by a final elongation step at 72 °C for 7 min. After amplification, samples were digested with DpnI (New England Biolabs, Beverly, MA) in order to simplify the extraction of only the newly amplified sequences from the PCR products. The digestion reaction was incubated at 37 °C for 3 hours with a total volume of 35 µL, which contained 30.5 µL of PCR products, 1 µL of DpnI and 3.5 µL of 10X buffer. Isolated products were subsequently used for cell transformation.

### Cell culture and luciferase assay of OF-ERV9-LTR

SW620 cells, derived from colorectal adenocarcinoma, were grown at 37 °C in a 5% (v/v) CO_2_ incubator in Rosewell Park Memorial Institute (RPMI) media (Gibco) supplemented with 10% (v/v) heat-inactivated foetal bovine serum (FBS) (Gibco) and 1% (v/v) antibiotic-antimycotic solution (Gibco, USA). Cells were grown to 70–80% confluency in 24-well plates before they were transferred into the relevant experimental medium, supplemented with only 10% (v/v) heat-inactivated FBS.

Transfection mixtures included 500 ng of the pGL-4.23 vector (Promega, USA) or a related construct containing either the OF-ERV-LTR sequences from chromosome 9, LTR sequence from chromosome 9 without the transcription binding site for Sox-5, LTR sequence from chromosome 9 without the transcription factor binding sites for Sox-5, GATA-1 and HNF-6, or a mutant form of the LTR sequence from chromosome 9 without any transcription binding sites. Transfection was performed using Lipofectamine 2000 (Invitrogen) as described in the manufacturer’s protocol. In addition, 100 ng of the pRL-TK plasmid (Promega, USA) was used as a control to normalize the transfection efficiency. Cells were lysed using a 1X buffer (Promega, USA) as provided and described in the manufacturer’s protocol, 24 hours after transfection and then stored at −80 °C for a minimum of 3 hours. Firefly and *Renilla* luciferase activities were assessed using the Dual-Luciferase® Reporter Assay System according to the manufacturer’s instructions.

### Relative expression analysis of *mkln1* and OF-miRNA-307 by qPCR amplification

Quantitative polymerase chain reaction (qPCR) primers for *mkln1* were designed using Primer3^52,53^ as follows: forward, 5ʹ-AGC ATC CAA ACA GCA CAG C-3ʹ and reverse, 5ʹ-CTC CGC CGA GTT AAA TAT CG-3ʹ. The amplification protocol was performed as follows: initial denaturation for 15 min at 95 °C; 45 cycles of 59 °C for 15 sec and 72 °C for 15 sec. Furthermore, a standard melting curve ramp ranging from 55 °C to 99 °C with a 1 °C rise on each step was performed. Universal GAPDH primers were used as a reference.

For OF-miRNA-307, each RNA sample was prepared in a reaction volume of 20 µL. The HB miR Multi Assay KitTM System I (HeimBiotek, Korea) was used for miRNA analysis in a 2-step process. For the initial cDNA synthesis, the HB_I Reverse Transcription (RT) Reaction Kit and its reagents were used according to manufacturer’s instructions. Reverse transcription polymerase chain reaction (RT-PCR) was then performed in a thermal cycler (Eppendorf, Hamburg, Germany), using the following conditions: 37 °C for 60 min (Step 1) followed by incubation at 95 °C for 5 min (Step 2) for one cycle and then held at 4 °C. The final product was stored at −20 °C until further use. For the second step, the HB_I Real-time PCR Master mix kit from the HB miR Multi Assay Kit™ System I was used according to the manufacturer’s suggestions using the Rotor-Gene Q system (QIAGEN, Hilden, Germany). The amplification protocol was performed as follows: initial denaturation for 15 min at 95 °C; 45 cycles of 95 °C for 10 sec and 60 °C for 40 sec, and a standard melting curve ramp ranging from 55 °C to 99 °C with a 1 °C rise on each step. Micro RNA U6 was used as a reference. The results were analysed as the relative expression ratio of the target miR-307 (5ʹ- ACA AAA CAC UUU UGG AGU UUC A -3′) to miRNA U6 using the comparative threshold method (2-ΔΔCt). All experiments were performed in triplicate and the mean values of the resulting relative expression ratios were used for analyses and the generation of charts.

### Co-transfection of psi-CHECK-2-*mkln1* 3′UTR and OF-miRNA-307 mimics

The schematic structure of *mkln1* shows that it encodes Muskelin N (yellow), while the Kelch3 (red) and Kelch1 (blue) proteins are included in the ORF of *mkln1*. Using the PCR primers forward: 5′-CGC CGG AAT TCT CGA GTC ACC ACT ACA TCC GTG GAG-3ʹ and reverse: 5ʹ-ATT GGA GCT CGA GCT CCC ACA GGA CAG TATG GAAG C-3′, the 3′-UTR region of the *mkln1* gene in olive flounder was amplified and then subsequently cloned into the XhoI cloning site of the psi-CHECK-2 vector (Promega). A forward primer targeting the psi-CHECK-2 vector and reverse primer targeting the *mkln1* gene were used to verify the orientation of the insert.

HT-29 cells were seeded in a 24-well dish at 4 × 10^4^ cells/well and grown to 60% confluency. After 24 hours, transfection was performed with Lipofectamine™ 2000 transfection reagent. OF-miRNA-307 miRNA mimics (Bioneer, Korea) were synthesized and then transfected into the HT-29 cell lines. Negative control miRNA was also included (Bioneer, Korea). The psi-CHECK-2-*mkln1*-3′UTR vector (100 ng) and miRNA mimics (0, 100, or 200 µM) were mixed with 50 µL of serum-free Opti-MEM (Gibco, USA). Then, 2 µL of Lipofectamine™ 2000 were added into each tube and mixed by tapping the tubes. The tubes were incubated at room temperature for 20 min. The mixture was then added to each well, following which the 24-well plate was rocked gently for 15 sec. The plate was then incubated for 24 hours in a 5% (v/v) CO_2_ humidified atmosphere at 37 °C. Firefly and Renilla luciferase activities were assessed using the Dual-Luciferase® Reporter Assay System according to the manufacturer’s instructions.

## Supplementary information

Supplementary informations

## Data Availability

The authors declare that all data supporting the findings of this study are available within the article or from the corresponding authors upon reasonable request.

## References

[1] Kitano T, Takamune K, Kobayashi T, Nagahama Y, Abe SI (1999). Suppression of P450 aromatase gene expression in sex-reversed males produced by rearing genetically female larvae at a high water temperature during a period of sex differentiation in the Japanese flounder (Paralichthys olivaceus). J. Mol. Endocrinol..

[2] Yoshinaga N (2004). Sexually dimorphic expression of a teleost homologue of Müllerian inhibiting substance during gonadal sex differentiation in Japanese flounder, Paralichthys olivaceus. Biochem. Biophys. Res. Commun..

[3] Fan Z (2014). Gonadal transcriptome analysis of male and female olive flounder (Paralichthys olivaceus). Biomed. Res. Int..

[4] Huang L (2015). *De Novo* assembly of the Japanese flounder (Paralichthys olivaceus) spleen transcriptome to identify putative genes involved in immunity. PLoS One.

[5] Isshiki I, Nishizawa T, Kobayashi T, Nagano T, Miyazaki T (2001). An outbreak of VHSV (viral haem-orrhagic septicemia virus) infection in farmed Japanese flounder Paralichthys olivaceus in Japan. Dis. Aquat. Organ.

[6] Moustafa E, Naota M, Morita T, Tange N, Shimada A (2010). Pathological Study on the Scuticociliatosis Affecting Farmed Japanese Flounder (Paralichthys olivaceus) in Japan. Journal of Veterinary Medical Science.

[7] Simora RM (2010). Molecular cloning and antiviral activity of IFN-b promoter stimulator-1 (IPS-1) gene in Japanese flounder, Paralichthys olivaceus. Fish Shellfish Immunol..

[8] Wang W (2014). Detection of alternative splice and gene duplication by RNA sequencing in Japanese flounder Paralichthys olivaceus. G3 (Bethesda).

[9] Lee HE, Ayarpadikannan S, Kim HS (2015). Role of Transposable elements in genomics rearrangement, evolution, gene regulation and epigenetics in primates. Genes & Genetic Systems.

[10] Kim HS (2012). Genomic impact, chromosomal distribution and transcriptional regulation of HERV elements. Mol. Cells.

[11] Gonzalez-Cao M (2016). Human endogenous retroviruses and cancer. Cancer Biol. Med..

[12] Johanning GL (2017). Expression of human endogenous retrovirus-K is strongly associated with the basal-like breast cancer phenotype. Sci. Rep..

[13] Akopov SB, Nikolaev LG, Khil PP, Lebedev YB, Sverdlov ED (1998). Long terminal repeats of human endogenous retrovirus K family (HERV-K) specifically bind host cell nuclear proteins. FEBS Letters.

[14] Vinogradova TV (2001). Solitary Human Endogenous Retroviruses-K LTRs Retain Transcriptional Activity *in Vivo*, the mode of Which Is Different in Different Cell Types. Virology.

[15] Hu T (2017). Long non-coding RNAs transcribed by ERV-9 LTR retrotransposon act in cis to modulate long-range LTR enhancer function. Nucleic Acids Research.

[16] Thompson PJ, Macfarlan TS, Lorincz MC (2016). Long terminal repeats: From parasitic elements to building blocks of the transcriptional regulatory repertoire. Molecular Cell.

[17] Ruda VM (2004). Tissue Specificity of enhancer and promoter activities of a HERV-K(HML-2) LTR. Virus Research.

[18] Choung EB, Rumi MAK, Soares MJ, Baker JC (2013). Endogenous retroviruses function as species-specific enhancer elements in the placenta. Nature Genetics.

[19] Nam GH (2016). Expression and promoter activity of endogenous retroviruses in the Olive flounder (Paralichthys olivaceus). Genes Genom..

[20] Bartel DP (2004). MicroRNAs: genomics, Biogenesis, mechanisms, and Function. Cell.

[21] Friedman RC, Farh KK-H, Burge CB, Bartel DP (2008). Most mammalian mRNAs are conserved targets of microRNAs. Genome Res..

[22] Ambros V (2003). A uniform system for microRNA annotation. RNA.

[23] Piriyapongsa J, Jordan IK (2007). A family of human microRNA genes from miniature inverted-repeat transposable elements. PLOS ONE.

[24] Piriyapongsa J, Mariño-Ramírez L, Jordan IK (2007). Origin and evolution of human microRNAs from transposable elements. Genetics.

[25] Crooks GE, Hon G, Chandonia JM, Brenner SE (2004). WebLogo: A sequence logo generator. Genome Research.

[26] Schneider TD, Stephens RM (1990). Sequence Logos: A new way to display consensus sequences. Nucleic Acids Res..

[27] Ashburner M (2000). Gene Ontology: tool for the unification of biology. Nature Genet..

[28] Huh JW (2008). Cooperative exonization of MaLR and AluJo elements contributed an alternative promoter and novel spliced variants of RNF19. Gene.

[29] Medstrand P, Landry JR, Mager DL (2001). Long terminal repeats are used as alternative promoters of the endothelin B receptor and apolopoprotein CI genes in humans. J. Biol. Chem..

[30] Wang T (2007). Species-specific endogenous retroviruses shape the transcriptional network of the human tumor suppressor protein p53. Proc. Natl. Acad. Sci. USA.

[31] Bourque G (2008). Evolution of the mammalian transcription factor binding repertoire via transposable elements. Genome Res..

[32] Sundaram V (2014). Widespread contribution of transposable elements to the innovation of gene regulatory networks. Genome Res..

[33] Jung YD (2017). Activity analysis of LTR12C as an effective regulatory element of the RAE1 gene. Gene.

[34] Wunderle VM, Critcher R, Ashworth A, Goodfellow PN (1996). Cloning and Characterization of SOX5, a New Member of the Human SOX Gene Family. Genomics.

[35] Lefebvre V (2010). The SoxD transcription factors – Sox5, Sox6, and Sox13 – are key cell fate modulators. Int. J. Biochem. Cell Biol..

[36] Liu CF, Lefebvre V (2015). The transcription factors SOX9 and SOX5/SOX6 cooperate genome-wide through super-enhancers to drive chondrogenesis. Nucleic Acids Research.

[37] Smits P (2001). The Transcription Factors L-Sox5 and Sox6 Are Essential for Cartilage Formation. Developmental Cell.

[38] Kamachi Y, Kondoh H (2013). Sox proteins: regulators of cell fate specification and differentiation. Development.

[39] Mata-Rocha M (2014). The transcription factors Sox5 and Sox9 regulate Catsper1 gene expression. FEBS Letters.

[40] Cheng JH, Pan DZC, Tsai ZTY, Tsai HK (2015). Genome-wide analysis of enhancer RNA in gene regulation across 12 mouse tissues. Scientific reports.

[41] Smalheiser NR, Torvik VI (2005). Mammalian microRNAs derived from genomic repeats. TRENDS in Genetics.

[42] Jo A, Lee HE, Kim HS (2019). Identification and expression analysis of a novel miRNA derived from ERV-E1 LTR in *Equus caballus*. Gene.

[43] Suzuki HI, Young RA, Sharp PA (2017). Super Enhancer-Mediated RNA Processing Revealed by Integrative MicroRNA Network Analysis. Cell.

[44] Xiao M (2017). MicroRNAs activate gene transcription epigenetically as an enhancer trigger. RNA Biology.

[45] Sperber G, Lövgren A, Eriksson N, Benachenhou F, Blomberg J (2009). RetroTector online, a rational tool for analysis of retroviral elements in small and medium size vertebrate genomic sequences. BMC Bioinformatics.

[46] Hall TA (1999). BioEdit: a user-friendly biological sequence alignment editor and analysis program for Windows 95/98/NT. Nucleic Acids Symposium Series.

[47] Kumar S, Stecher G, Tamura K (2016). MEGA7: Molecular Evolutionary Genetics Analysis version 7.0 for bigger datasets. Molecular Biology and Evolution.

[48] Rehmsmeier M, Steffen P, Hochsmann M, Giegerich R (2004). Fast and effective prediction of microRNA/target duplexes. RNA.

[49] Kim D, Park E, Kim J (1982). Karyotype of nine species of the Korean catfishes (Teleostomi:Siluriformes). Korean J. Genet..

[50] Waminal NE, Kim HH (2012). Dual-color FISH karyotype and rDNA distribution analyses on four Cucurbitaceae species. Horticulture, Environment, and Biotechnology.

[51] Matoba H, Mizutani T, Nagano K, Hoshi Y, Uchiyama H (2007). Chromosomal study of lettuce and its allied species (Lactuca spp., Asteraceae) by means of karyotype analysis and fluorescence *in situ* hybridization. Hereditas.

[52] Untergasser A (2012). Primer3 - new capabilities and interfaces. Nucleic Acids Research.

[53] Koressaar T, Remm M (2007). Enhancements and modifications of primer design program Primer3. Bioinformatics.

