# Supplementary material for: Characterization of the Long Terminal Repeat of the Endogenous Retrovirus-derived microRNAs in the Olive Flounder
Source: Sci Rep. 2019 Sep 30;9:14007. doi: 10.1038/s41598-019-50492-7 (PMC6768988; doi:10.1038/s41598-019-50492-7)
Supplement: Supplementary file 1 — Supplementary informations [file 41598_2019_50492_MOESM1_ESM.pdf]

Characterization of the Long Terminal Repeat of the Endogenous Retrovirus-derived microRNAs in the Olive Flounder

Hee-Eun Lee[1,2,+], Ara Jo[1,2,+], Jennifer Im[1], Hee-Jae Cha[3], Woo-Jin Kim[4], Hyun Hee Kim[5,6], Dong-Soo Kim[7], Won Kim[8], Tae-Jin Yang[9], Heui-Soo Kim[1, 2]*

[1] Department of Biological Sciences, College of Natural Sciences, Pusan National University, Busan 46241, Republic of Korea

[2] Institute of Systems Biology, Pusan National University, Busan 46241, Republic of Korea

[3] Department of Parasitology and Genetics, Kosin University College of Medicine, Busan 49267, Republic of Korea

[4] Biotechnology Research Divisions, National Fisheries Research and Development Institute, Busan 46083, Republic of Korea

[5] Departments of Life Science, Sahmyook University, Seoul 01795, Republic of Korea

[6] Chromosome Research Institute, Sahmyook University, Seoul 01795, Republic of Korea

[7] Department of Marine Bio-Materials & Aquaculture, Pukyong National University, Busan 48513, Republic of Korea

[8] School of Biological Sciences, Seoul National University, Seoul 08826, Republic of Korea

[9] Department of Plant Science, Plant Genomics and Breeding Institute, Research Institute for Agriculture and Life Sciences, College of Agriculture and Life Sciences, Seoul National University, Seoul, 151-921, Republic of Korea

+ These authors contributed equally to this work

[*] Correspondence to: Prof. Heui-Soo Kim

Department of Biological Sciences

College of Natural Sciences

Pusan National University

Busan 46241

Republic of Korea

Fax: +82 51 581 2962

Tel: +82 51 510 2259

E-mail: khs307@pusan.ac.kr

Suppl Fig. 1

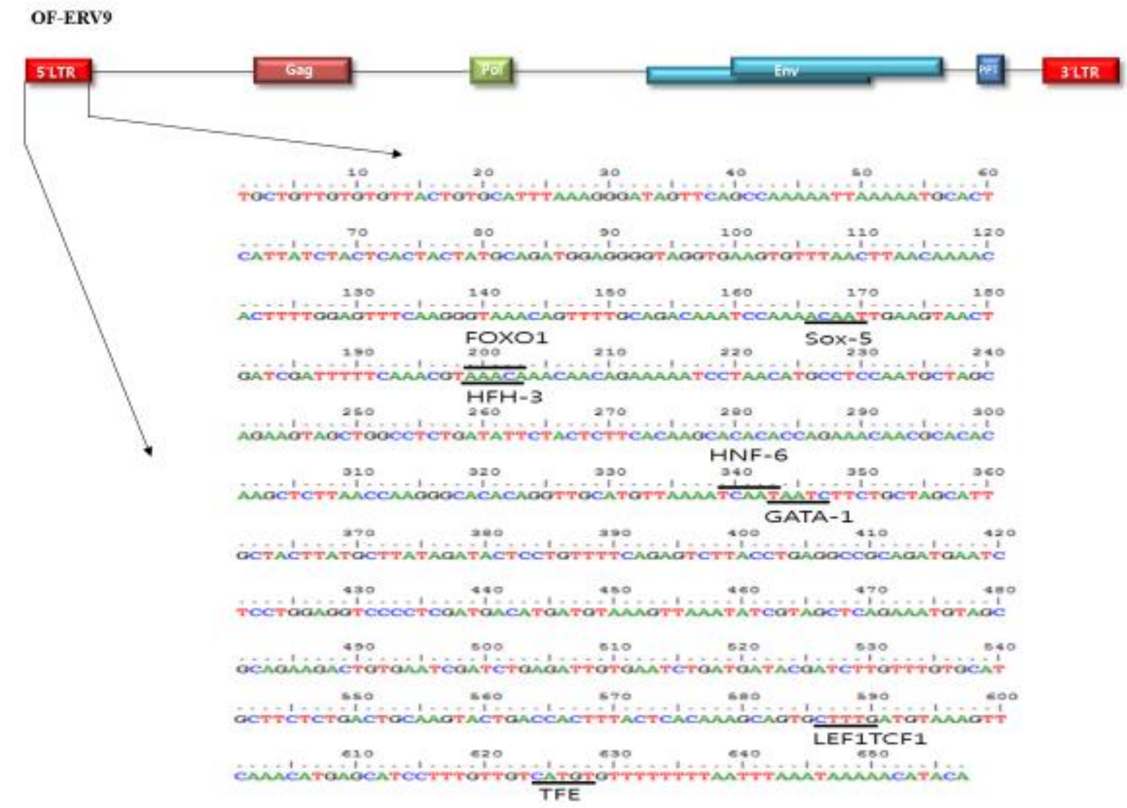

Suppl. Fig. 1

Schematic structure of OF-ERV9 and its predicted transcription factor binding sites, focusing on the 5′ LTR region. Only binding sites with a high threshold for core match and matrix match were selected for further study.

Suppl Fig. 2

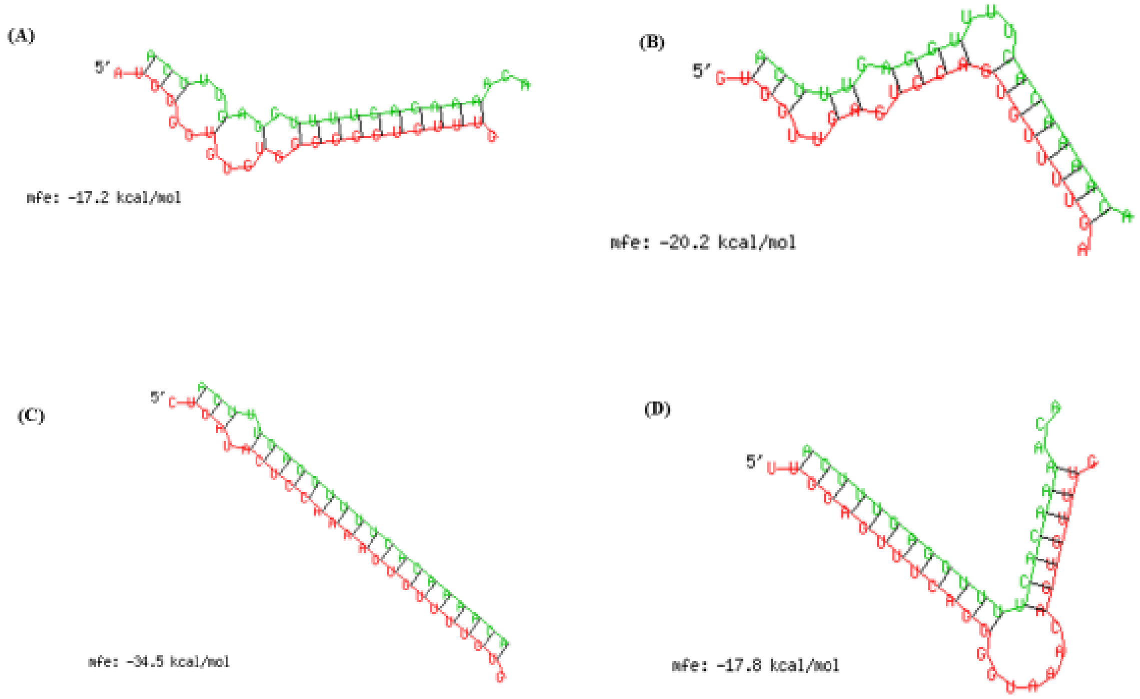

Suppl. Fig. 2

The RNAhybrid structure of OF-miRNA-307 and its related genes. Red represents OF-miRNA-307 and green represents each related gene.

(A) OF-miRNA-307 and LOC109644478, having an energy scale of -17.2 kcal/mol.

(B) OF-miRNA-307 and *mkln1*, having an energy scale of -20.2 kcal/mol.

(C) OF-miRNA-307 and LOC109636349, having an energy scale of -34.5 kcal/mol.

(D) OF-miRNA-307 and *slc37a3*, having an energy scale of -17.8 kcal/mol.

Suppl Fig. 3

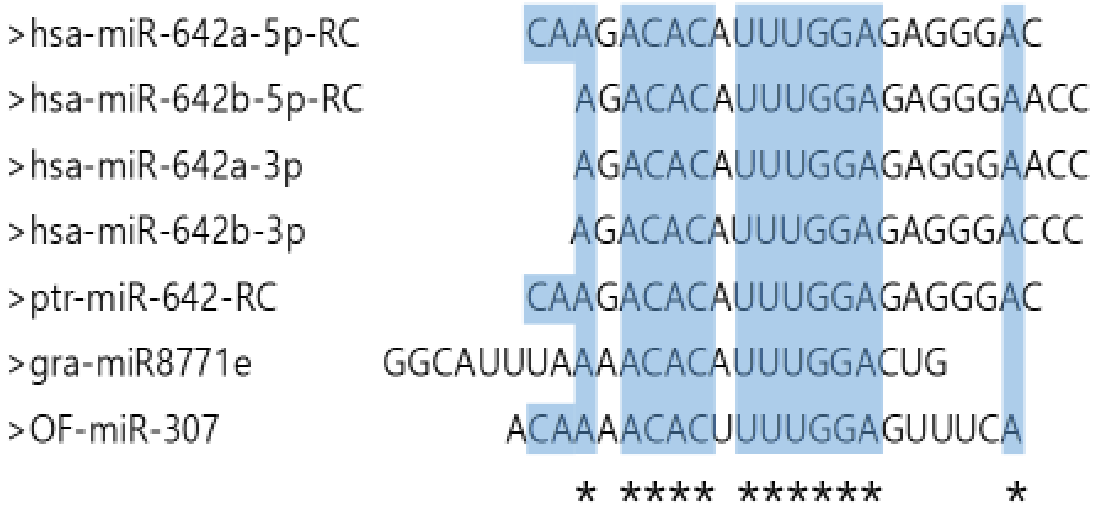

Suppl. Fig. 3

Sequence alignments of OF-miRNA-307 and its related miRNAs. Six similar miRNAs were detected. Four of these are human based miRNA, as indicated by the ‘hsa' prefix, and all six represent miRNA 642 family. The asterisks and blue shading indicate the conserved regions.

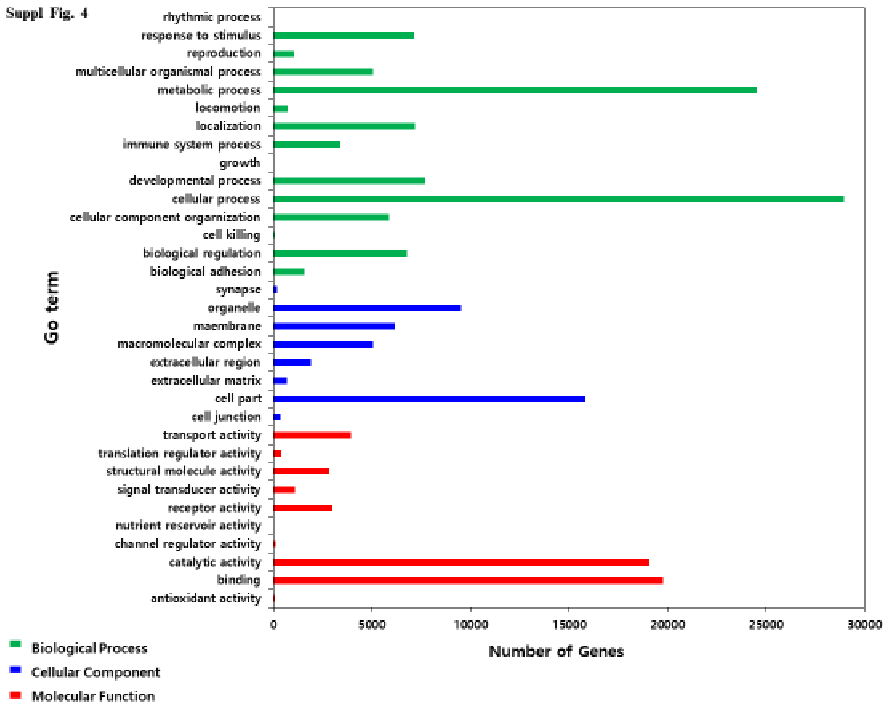

Suppl. Fig. 4

The gene ontology results of hsa-miR-642a-5p, which is the closely related miRNA to OF-miRNA-307. has-miR-642a-5p was examined since OF-miRNA-307 is not yet registered in the miR-base system. Red indicates molecular function, blue for cellular component, and green for biological process. Metabolic process and cellular process are the most strongly represented, followed by catalytic activity and binding.
